# Design and Validation of a Single-SOI-Wafer 4-DOF Crawling Microgripper

**DOI:** 10.3390/mi10060376

**Published:** 2019-06-05

**Authors:** Matteo Verotti, Alvise Bagolini, Pierluigi Bellutti, Nicola Pio Belfiore

**Affiliations:** 1 Department of Mechanical, Energy, Management and Transportation Engineering, University of Genoa, 16145 Genoa, Italy; 2 Micro Nano Facility, Fondazione Bruno Kessler, 38123 Trento, Italy; bagolini@fbk.eu (A.B.); bellutti@fbk.eu (P.B.); 3 Department of Engineering, Roma Tre University, 00146 Rome, Italy; nicolapio.belfiore@uniroma3.it

**Keywords:** microgripper, microstage, lab-on-chip, flexure, conjugate surface flexure hinge (CSFH), silicon-on-insulator (SOI) wafer, deep-reactive ion etching (DRIE)

## Abstract

This paper deals with the manipulation of micro-objects operated by a new concept multi-hinge multi-DoF (degree of freedom) microsystem. The system is composed of a planar 3-DoF microstage and of a set of one-DoF microgrippers, and it is arranged is such a way as to allow any microgripper to crawl over the stage. As a result, the optimal configuration to grasp the micro-object can be reached. Classical algorithms of kinematic analysis have been used to study the rigid-body model of the mobile platform. Then, the rigid-body replacement method has been implemented to design the corresponding compliant mechanism, whose geometry can be transferred onto the etch mask. Deep-reactive ion etching (DRIE) is suggested to fabricate the whole system. The main contributions of this investigation consist of (i) the achievement of a relative motion between the supporting platform and the microgrippers, and of (ii) the design of a process flow for the simultaneous fabrication of the stage and the microgrippers, starting from a single silicon-on-insulator (SOI) wafer. Functionality is validated via theoretical simulation and finite element analysis, whereas fabrication feasibility is granted by preliminary tests performed on some parts of the microsystem.

## 1. Introduction

A large variety of applications has recently required micro-scale devices to perform gripping and positioning tasks. The general interest in developing micromanipulation systems has been evidenced by more than a hundred different microgrippers that have been proposed in the literature [1,2]. For example, electrostatically-actuated microgrippers have been presented to test different materials sets, such as Si/SiO${}_{2}$ and polysilicon/Si${}_{3}$N${}_{4}$ [3], and for the manipulation of glass microspheres [4] or SiO${}_{2}$ nanoparticles [5].

Microgrippers are also widely used for biomedical applications. A hot-and-cold-arm electrothermally-actuated microgripper has been designed for the deformability study of human red blood cells [6]. Further investigations have been conducted focusing on actuation systems for bio-MEMS (microelectromechanical systems) applications, in viscous dielectric media [7,8,9] or underwater [10,11].

MEMS-based microgrippers, equipped with rotary comb-drive actuators, have been presented to characterize the mechanical properties of cells [12,13,14]. Further applications of microgrippers could consists of in vitro simulation of basic surgical operations, by testing and analyzing the microgripper–tissue interactions. This possibility paves the way to operations that appears currently possible in a series of surgical applications, such as endoluminal [15,16,17], minimally-invasive [18,19,20], or gastrointestinal surgery [21].

Many of the presented microgrippers have a limited number of degrees of freedom (DoFs), usually only one. In fact, at the microscale, the design of multi-DoFs gripping systems is quite challenging from several points of view, such as kinematic synthesis, the definition of proper actuation and sensing schemes, and fabrication processes. For this reason, microgrippers are often used in combination with micro-/nano-positioning stages. These devices have been used in many research fields with various applications, such as cell injection [22] and mask alignment [23]. In addition to conventional positioning systems based on rigid-body mechanisms, compliant microstages have been widely designed and implemented [24,25,26,27] because of their features, such as no backlash, low cost, and vacuum compatibility [28]. MEMS-based technologies, including deep reactive-ion etching (DRIE) of silicon-on-insulator (SOI) wafers, have been used to fabricate compliant, monolithic, silicon structures. In particular, SOI wafers are composed of two silicon layers, namely, the *handle* and the *device* layers, and both of them can be etched in order to fabricate complex structures. For example, this technique has been applied to develop a silicon capacitive multiple force and torque sensor [29] and a self-aligned electrostatic vertical comb-drive [30].

In this paper, a new concept device is proposed to simplify the manipulation tasks at the microscale and to overcome the technical complication of an arrangement composed of two separate and cooperating tools: the gripper and the stage. The system consists of a 3-DoF planar platform, based on the 3-RRR (revolute joint) parallel linkage [31], and of a set of 1-DoF microgrippers. The combination of the two different motions, of the platform and of the gripper, gives rise to a 4-DoF device. The system is designed to be fabricated starting from a single SOI wafer with DRIE technology, and a specific process flow is designed to guarantee the fabrication of both the grippers and the stage. The forward kinematics problem is solved for the 3-RRR rigid-body platform, whereas finite element simulations are carried out for the corresponding compliant mechanism. Fabrication feasibility is also verified by means of preliminary tests.

## 2. Design of a New 4-DoF Microsystem

In this section, the steps followed to design the micromanipulation system and its working principle are described.

### 2.1. Topology

**Figure 1:**
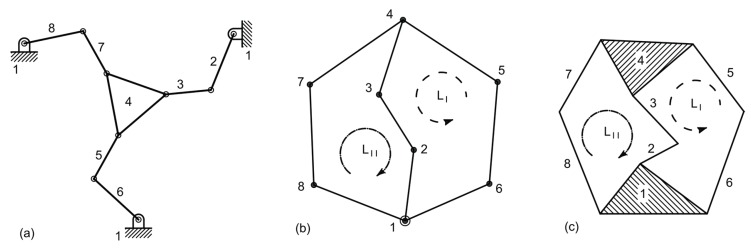
Functional representation of the adopted 3-DoF platform (**a**) together with its corresponding graph (**b**) and kinematic chain (**c**).

The first subsystem consists a 3-DoF plane parallel platform suspended on three legs, each one having three revolute joints (3-RRR platform). A possible configuration for the system is represented in Figure 1a, with the adopted nomenclature. Platform 4 is moved by the three dyads 2-3, 6-5, and 8-7. The corresponding graph representation, reported in Figure 1b, reveals that the plane mechanism has two independent loops, $L_{IND}=2$, which can be identified also from the sketch of the kinematic chain presented in Figure 1c. Loops $L_{I}=1\;-\;6\;-\;5\;-\;4\;-\;3\;-\;2\;-\;1$ and $L_{II}=1\;-\;8\;-\;7\;-\;4\;-\;3\;-\;2\;-\;1$ are not uniquely identified because the outer loop 1-6-5-4-7-8-1 could be used in place of either $L_{I}$ or $L_{II}$. This third loop cannot be used for the sake of the kinematic analysis because it is dependent on the other two. This is also clear from Euler equation:

(1) $$L_{IND}=m-\ell +1=2\;,$$

m=9 and ℓ=8 being the number of kinematic pairs and links, respectively.

By using Grubler’s (simplified) topological formula:

(2) $$\begin{array}{c} F=3\left(\ell -1\right)-2m\; \end{array}$$

the number *F* of DoFs are immediately calculated as equal to three, and so, three input parameters will be needed to identify a configuration. In this investigation, the input links are those that are adjacent to the frame 1, namely, 2, 6, and 8, and so, their angular positions are treated as independent variables. The topological structure of a 3-RRR plane platform is represented by the graph or the kinematic chain reported in Figure 1b,c, respectively. An ordinary mechanism can be obtained, as the one represented in Figure 1a. This mechanism can be designed by means of the methods of kinematic synthesis [32,33].

The second subsystem consists of a set of two-finger grippers, each finger rotating about a fixed axis. The fingers are not allowed to contact the platform because they work on different, but parallel planes.

### 2.2. Working Principle

**Figure 2:**
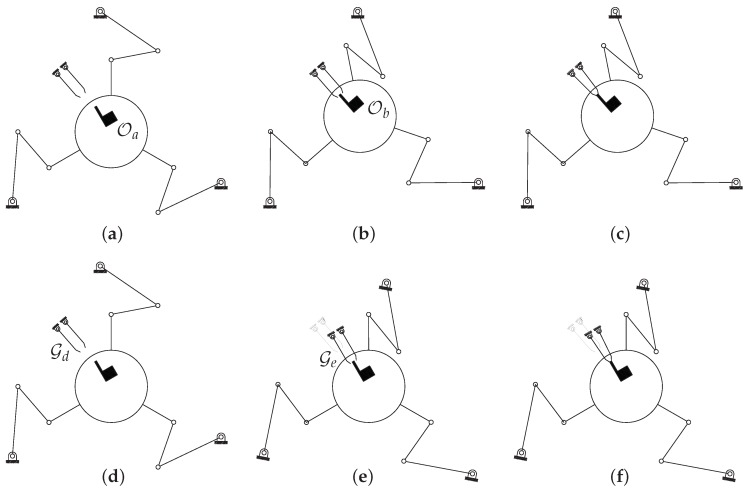
Absolute and relative motions sequences, from (**a**–**c**) and from (**d**–**f**), respectively.

The working principle is described in Figure 2, which illustrates the sequence (**a**)-(**b**)-(**c**) for the platform absolute motion, and the sequence (**d**)-(**e**)-(**f**) for the relative motion of the gripper with respect to the platform. Figure 2a depicts the starting configuration. By actuating the three electrostatic motors, the pose reported in Figure 2b is obtained.

This figure also shows that both translations (2 DoFs) and rotations (1 DoF) can be assigned (full mobility in the plane) to the object to be manipulated. In fact, the object moves from pose $O_{a}$ to $O_{b}$. Finally, the object is gripped by the fingers, as reported in Figure 2c. By using a reference frame attached to the mobile platform, the relative motion can be described as in the sequence (**d**)-(**e**)-(**f**). Figure 2d shows again the starting position, whereas Figure 2e represents the motion of the gripper relative to the object. Therefore, in the relative motion, the gripper crawls from pose $G_{d}$ to $G_{e}$. Finally, the gripper grasps the object as illustrated in Figure 2f.

### 2.3. Application of the Rigid-Body Replacement Method

**Figure 3:**
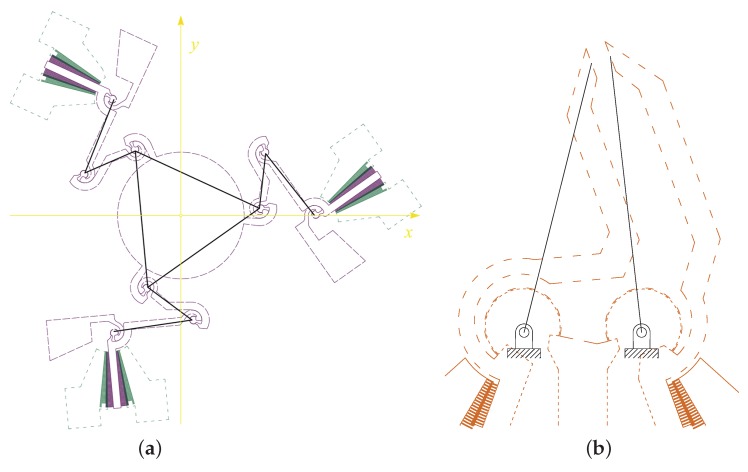
Pseudo-rigid body model (solid line) and corresponding compliant mechanism (dashed line) of the stage (**a**) and of the microgripper (**b**).

Once the ordinary mechanism has been synthesized, a compliant mechanism can be obtained by building a mechanism with lumped compliance. The mechanism with an ordinary kinematic pair is also known as a pseudo-rigid body model (PRBM) [34,35,36], such as the ones reported in solid line in Figure 3a,b, representing the stage and the gripper, respectively. Starting from the PRBMs, it is possible to obtain the corresponding compliant mechanisms as the ones represented in dashed line.

This procedure is not unique. In the case under study, a criterion has been used according to which the center of the elastic weights of the flexible circular arc beam is positioned in correspondence to the center of relative rotation between two subsequent rigid bodies.

### 2.4. Implementation

**Figure 4:**
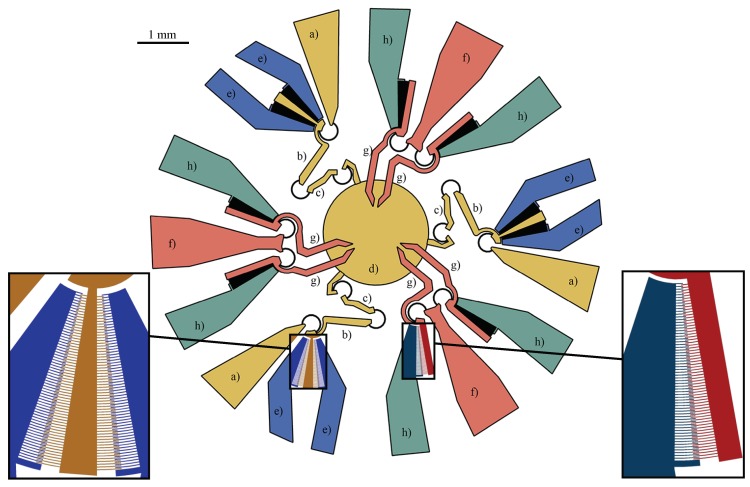
A top view of the whole microsystem composed of three anchored electrodes (**a**), three moving arms (**b**), three coupler arms (**c**), a platform (**d**), three anchored electrodes (**f**), six jaws (**g**), and six anchored electrodes (**h**). On the left, a magnified inset showing the bi-directional rotary comb-drive of the stage. On the right, the detail of the microgripper comb-drive.

An overall view of the system is depicted in Figure 4. With reference to the labels used in this figure, a round platform (**d**) is driven by three RRR legs, each one composed of two binary links (**b**) and (**c**) arranged as a series and an anchored electrode (**a**). A link (**b**) has the same electric potential as the adjacent electrode (**a**), and it is mechanically connected to two symmetric rotating finger sets, which are interdigitated with two more finger sets anchored to electrodes (**e**) to provide bilateral rotations. The different electric potential values on (**e**)-type electrodes define the rotations of the moving arms (**b**) and, therefore, the motion of the platform (**d**), which has three DoFs in the plane. Each jaw (**g**) of the gripper rotates around the center of the elastic weights of the curved flexure through which the jaw is suspended by the anchored electrode (**f**). Each jaw is also connected to a rotating set of fingers that are interdigitated with one more anchored finger set (**h**).

**Figure 5:**
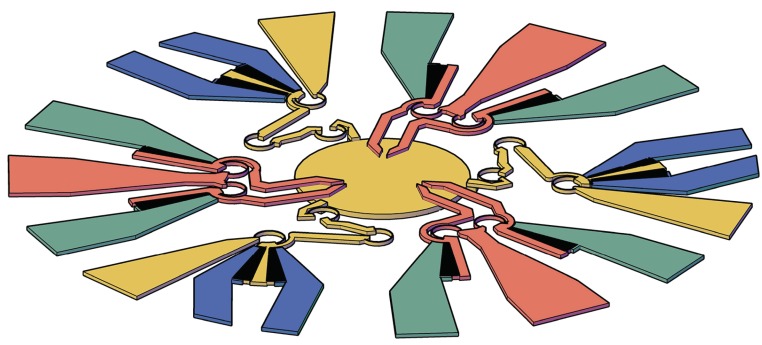
Perspective view of the proposed microsystem.

The perspective view depicted in Figure 5 may be helpful to distinguish the different working planes:

- an upper device layer, which includes all the elements except the platform;
- a lower layer, which consists of the suspended platform;
- an intermediate oxide layer, which provides connections between the upper and lower layers.

The central platform (**d**) is hanging from the ceiling of three clamps obtained on the end parts of the type (**c**) coupler arms. Contacts among the overlapping grippers and platform are avoided thanks to the fabrication process, which will be described in Section 5.

Alternative geometries for the compliant 3-RRR plane platform can be obtained by replacing the constant-curvature flexures with conjugate surface flexure hinges (CSFHs) [37,38,39,40]. The adoption of the CSFHs can be useful to increase the accuracy of the planned motion [41,42], but it implies the occurrence of possible contacts among the conjugate profiles.

As illustrated in Figure 4, the platform motion is governed by rotary bidirectional comb-drives, whereas the gripping task is provided by rotary unidirectional comb-drives, the latter tested in a previous work [38]. For the sake of completeness, it is worth noting that the tested comb-drives have been built with the same technological process considered in this investigation (DRIE), with the following design parameters: number of fingers n=100, fingers gap g=3μm, fingers width w=3μm, actuation potential equal to 17 V. Discussions about some problems that could affect the electrostatic actuators to be employed, such as position accuracy and pull-in effect, are also provided in [38].

## 3. Simulation via a Theoretical Approach

The platform under study was conceived in such a way that the lengths of Links 2, 6, and 8 are all equal to a given value *a*. Similarly, the lengths of links 3, 5, and 7 are all equal to *b*. Finally, the platform edge lengths are equal to *c* and the fixed distance between any two fixed revolute pairs is *d*.

**Figure 6:**
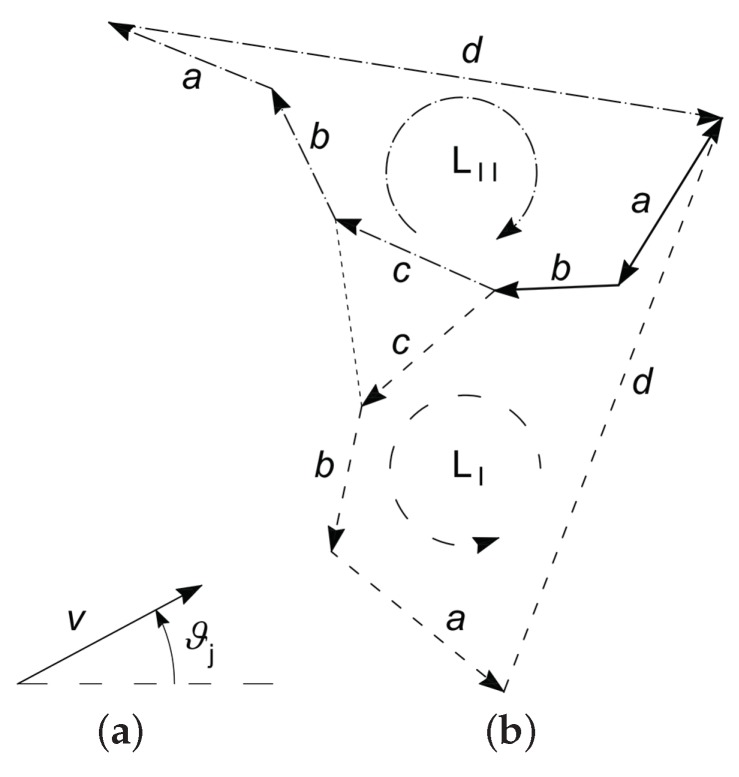
According to the adopted nomenclature, a vector *v* direction is defined by its angle $\vartheta_{j}$ measured w.r.t. the horizontal direction (**a**); this convention has been used to characterize the orientation of the vectors that compose the two loops $L_{I}$ and $L_{II}$ (**b**).

Using the complex number representation of plane vectors and assuming the nomenclature reported in Figure 6, the closed-loop equations can be written as:

(3) $$be^{I\theta_{3}}-be^{I\theta_{5}}+ce^{I\theta_{4}}\left(\frac{1}{2}+\frac{1}{2}I\sqrt{3}\right)+G=0\;,$$

(4) $$be^{I\theta_{3}}-be^{I\theta_{7}}+ce^{I\theta_{4}}+H=0\;,$$

where $I=\sqrt{-1}$, with

(5) $$G=a\left(e^{I\theta_{2}}-e^{I\theta_{6}}\right)+d\left(\frac{1}{2}\sqrt{3}+\frac{1}{2}I\right)\;,$$

(6) $$H=a\left(e^{I\theta_{2}}-e^{I\theta_{8}}\right)+d\left(\frac{1}{2}\sqrt{3}-\frac{1}{2}I\right)\;.$$

Rearranging Equations (3) and (4), the two unit module vectors:

(7) $$e^{I\theta_{4}}=\frac{2\left(be^{I\theta_{5}}+ae^{I\theta_{6}}-Id-be^{I\theta_{7}}-ae^{I\theta_{8}}\right)}{c\left(-1+I\sqrt{3}\right)}$$

and:

(8) $$e^{I\theta_{3}}=\frac{-2be^{I\theta_{5}}+be^{I\theta_{7}}+Ibe^{I\theta_{7}}\sqrt{3}-H-IH\sqrt{3}+2G}{b\left(-1+I\sqrt{3}\right)}$$

are obtained. Hence, by imposing that the right-hand sides of (8) and (7) have a unit value, two constraint equations, which are

(9) $$\begin{array}{cc} \psi_{1} & =\frac{1}{c^{2}}\left(-2\;b\cos\theta_{5}a\cos\theta_{8}-2a\cos\theta_{6}b\cos\theta_{7}+\right.+2b\cos\theta_{7}a\cos\theta_{8}-2b\sin\theta_{5}a\sin\theta_{8}+\\ & -2a\sin\theta_{6}b\sin\theta_{7}++2b\sin\theta_{7}a\sin\theta_{8}-c^{2}+2\;a^{2}-2\;a\sin\theta_{6}d+d^{2}+\\ & -2\;a^{2}\cos\theta_{6}\cos\theta_{8}-2\;b^{2}\cos\theta_{5}\cos\theta_{7}+2\;b\cos\theta_{5}a\cos\theta_{6}+2\;b^{2}+2\;b\sin\theta_{5}a\sin\theta_{6}+\\ & -2\;b^{2}\sin\theta_{5}\sin\theta_{7}-2\;b\sin\theta_{5}d+-2\;a^{2}\sin\theta_{6}\sin\theta_{8}+2\;da\sin\theta_{8}+2\;db\sin\theta_{7}=0 \end{array}$$

and

(10) $$\begin{array}{cc} \psi_{2} & =\frac{1}{b^{2}}\left(\sqrt{3}a^{2}\sin\theta_{8}\cos\theta_{6}+b^{2}+3\;a^{2}+\right.-b^{2}\sin\theta_{5}\sin\theta_{7}-a^{2}\cos\theta_{6}\cos\theta_{8}-b^{2}\cos\theta_{5}\cos\theta_{7}+\\ & -\sqrt{3}b^{2}\sin\theta_{5}\cos\theta_{7}-b\sin\theta_{7}a\sin\theta_{2}-b\cos\theta_{7}a\cos\theta_{2}+-a^{2}\sin\theta_{6}\sin\theta_{8}-a\cos\theta_{6}b\cos\theta_{7}+\\ & +2\;b\cos\theta_{7}a\cos\theta_{8}-a\sin\theta_{6}b\sin\theta_{7}+2\;b\cos\theta_{5}a\cos\theta_{6}-b\cos\theta_{5}a\cos\theta_{8}+\\ & +2\;b\sin\theta_{5}a\sin\theta_{6}-\sqrt{3}b\sin\theta_{5}a\cos\theta_{8}-\sqrt{3}a\sin\theta_{6}b\cos\theta_{7}+\sqrt{3}b\sin\theta_{7}a\cos\theta_{6}+\\ & +\sqrt{3}a\sin\theta_{8}b\cos\theta_{5}+\sqrt{3}b\sin\theta_{5}a\cos\theta_{2}-a^{2}\cos\theta_{8}\cos\theta_{2}-a^{2}\sin\theta_{6}\sin\theta_{2}-a^{2}\sin\theta_{2}\sin\theta_{8}+\\ & +\sqrt{3}a^{2}\cos\theta_{8}\sin\theta_{2}-b\sin\theta_{5}a\sin\theta_{8}+2\;b\sin\theta_{7}a\sin\theta_{8}-\sqrt{3}a^{2}\sin\theta_{2}\cos\theta_{6}-\sqrt{3}a^{2}\sin\theta_{8}\cos\theta_{2}+\\ & -b\cos\theta_{5}a\cos\theta_{2}-b\sin\theta_{5}a\sin\theta_{2}+\sqrt{3}a^{2}\cos\theta_{2}\sin\theta_{6}+\sqrt{3}b^{2}\sin\theta_{7}\cos\theta_{5}+-a^{2}\cos\theta_{6}\cos\theta_{2}+\\ & -\sqrt{3}b\cos\theta_{5}a\sin\theta_{2}-\sqrt{3}b\sin\theta_{7}a\cos\theta_{2}++\sqrt{3}b\cos\theta_{7}a\sin\theta_{2}-\sqrt{3}a^{2}\sin\theta_{6}\cos\theta_{8}=0\;, \end{array}$$

are obtained, where angles $\theta_{3}$ and $\theta_{4}$ are eliminated. As a consequence, only $\theta_{5}$ and $\theta_{7}$ remain dependent variables, since $\theta_{6}$, $\theta_{8}$, and $\theta_{2}$ are input values. Although such a system of equations is not linear, it can be solved by means of elementary numerical procedures, for example by means of the Newton–Raphson method. The angles $\theta_{3}$ and $\theta_{4}$ can be easily calculated through Equations (7) and (8), once the system (9) and (10) has been solved w.r.t. $\theta_{5}$ and $\theta_{7}$.

Finally, the tip position *p* is given by:

(11) $$p=ae^{I\theta_{2}\left(t\right)}+be^{I\theta_{3}\left(t\right)}+\frac{1}{2}c\sqrt{3}e^{I\theta_{4}\left(t\right)}\left(\frac{1}{2}\sqrt{3}+\frac{1}{2}I\right)+\frac{1}{2}\sqrt{3}\;.$$

**Figure 7:**
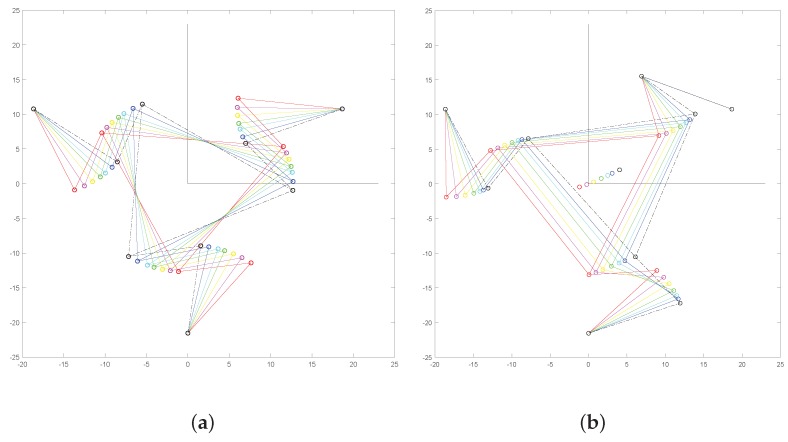
Two sequences of consecutive poses in the case of a large rotation (**a**) and translation (**b**) of the platform, obtained by means of the theoretical approach.

Figure 7 shows the results of iterated position analysis in the case of equal (**a**) or emisymmetric (**b**) rotations of the input links. The latter motion refers to the case for which one input link is kept in the initial position and the other two links rotate with equal magnitude and opposite direction. The simulations were performed considering large rotations for the input links. The figure shows that the platforms rotates in the case of (**a**) or translates approximately in the case of (**b**). The two cases will be discussed in detail in Section 4.1, Section 4.2 and Section 6.1.

## 4. Simulation via Finite Element Analysis

**Figure 8:**
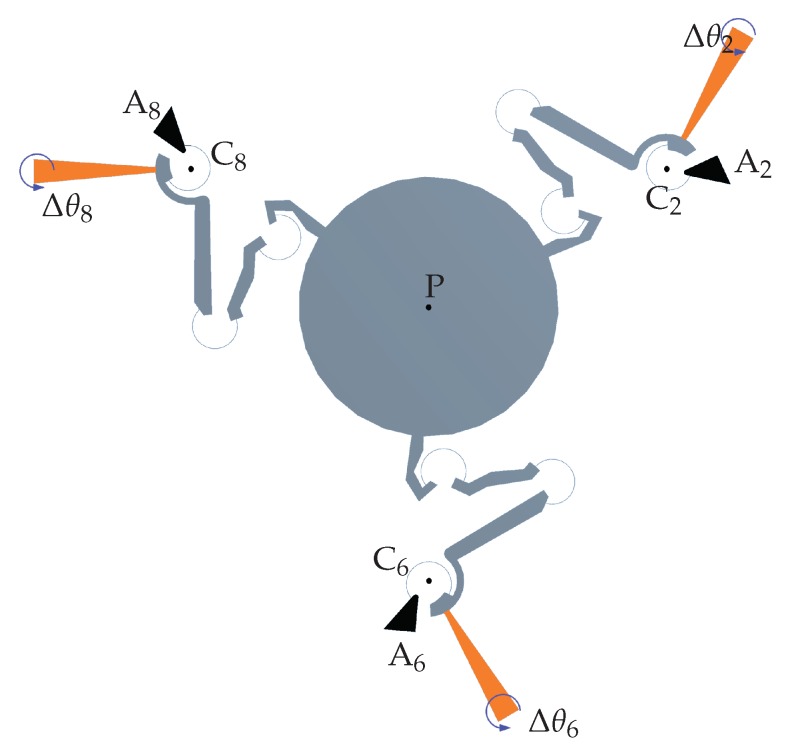
Finite element simulations’ setup: fixed supports $A_{i}$ (black regions) and assigned rotations $\Delta \theta_{i}$ to the comb-drive rotors (orange regions) around the corresponding center $C_{i}$, i=2,6,8.

To analyze the planar motion of the platform, finite element simulations were performed with the commercial software ANSYS © (v. 18.1), considering the anisotropic formulation of elasticity for silicon [43] and nonlinearity due to large deflections. With reference to Figure 8, the fixed supports $A_{i}$ have been introduced in the anchored parts (black regions). To model the rotary motion of each actuators, a set of rotations have been assigned to each comb-drive rotor with respect to its corresponding center $C_{i}$. The rotations of the independent links i=2,6,8 were measured with respect to their neutral configurations ${\hat{\theta }}_{i}$, and therefore, they will be identified by $\Delta \theta_{i}=\theta_{i}\left(t\right)-{\hat{\theta }}_{i}$. The generated mesh was composed of 17,775 nodes and 73,958 elements, locally refined for the flexible elements. Two sets of simulations have been performed, as described in the next subsections.

### 4.1. Uniform Rotations of the Independent Links

**Figure 9:**
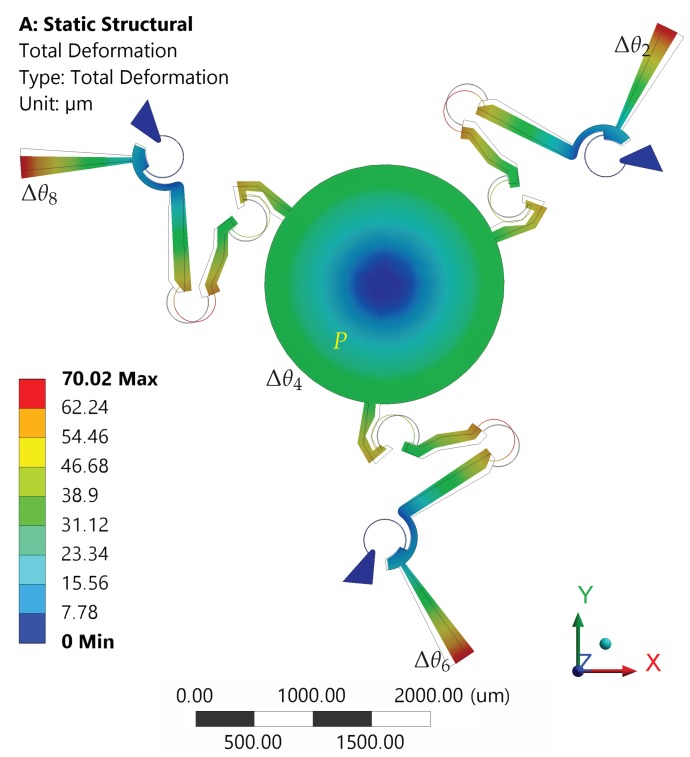
Structure in neutral (wireframe) and deformed (solid) configurations for $\Delta \theta_{2}\;=\;\Delta \theta_{6}\;=\;\Delta \theta_{8}\;=\;3$°.

In the first set of simulations, equal rotations have been assigned to each rotor ($\Delta \theta_{2}\;=\;\Delta \theta_{6}\;=\;\Delta \theta_{8}$), from 0° to 3° with steps of 0.25°. Figure 9 shows the neutral configuration and the deformed one for the case $\Delta \theta_{2}\;=\;\Delta \theta_{6}\;=\;\Delta \theta_{8}\;=\;\Delta \theta \;=\;3$°.

**Figure 10:**
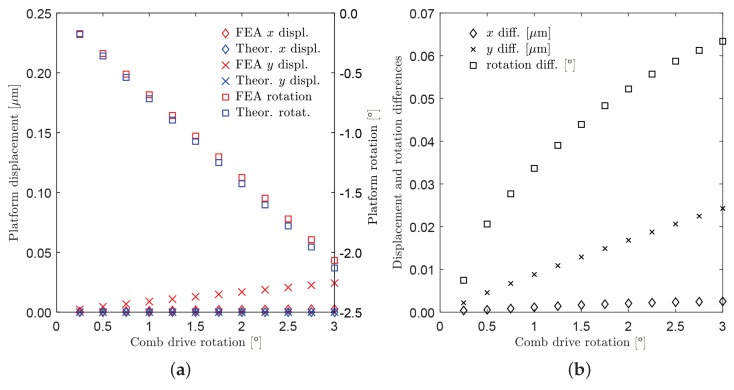
Case of uniform rotations ($\Delta \theta_{2}\;=\;\Delta \theta_{6}\;=\;\Delta \theta_{8}\;=\;\Delta \theta$). (**a**) Displacements of the platform tip along the *x*-axis and *y*-axis (diamond and cross markers, left-hand side axis) and rotations of the platform (square markers, right-hand side axis) as a function of Δθ; red and blue colors refer to FEA and theoretical simulations, respectively. (**b**) Absolute differences between FEA and theoretical simulated values (see the legend).

The platform rotations and the tip displacements (point P), with respect to the assigned comb-drive rotations Δθ, are reported in Figure 10a. When the maximum input rotation Δθ=3° was applied, the platform rotation $\Delta \theta_{4}$ reached a value of about 2°, while the platform center tip presented a very limited displacement from the original position. In fact, for this case, the rotational motion was prevailing over translational motion. Figure 10b represents the absolute differences among the results obtained by means of FEA and theoretical simulation. For example, for a given input rotation Δθ=3° of the moving arms, the methods predicted the platform rotations $\Delta \theta_{4}$ with a difference of about 0.06°, while the maximum difference in *x* or *y* linear displacements was less than 0.03 μm.

### 4.2. Emisymmetric Rotations of the Independent Links

In the second set, rotations with equal magnitude, but opposite signs have been assigned to rotors 2 and 6 ($\Delta \theta_{2}\;=\;-\Delta \theta_{6}$), from 0°–3° with steps of 0.25°. No rotations have been assigned to the third rotor ($\Delta \theta_{8}=0$).

**Figure 11:**
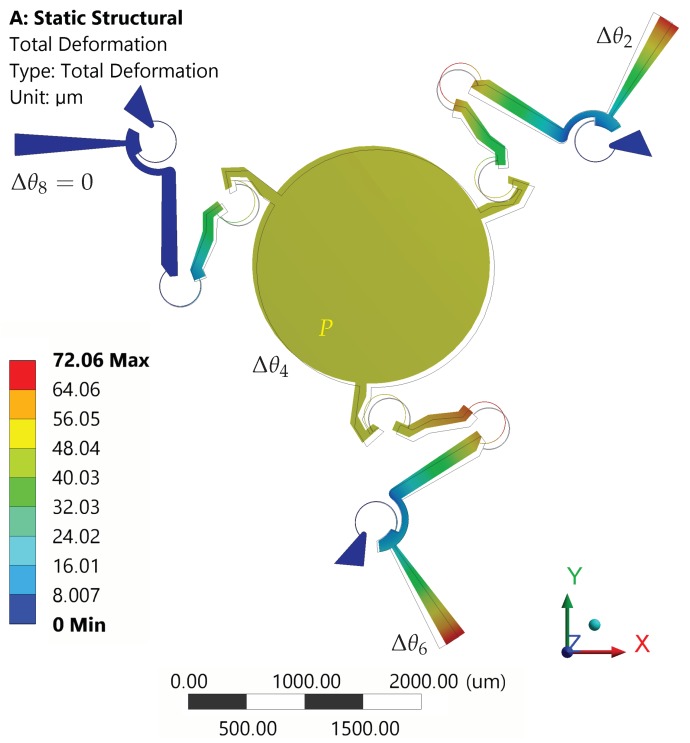
Structure in neutral (wireframe) and deformed (solid) configurations for $\Delta \theta_{2}\;=\;-\Delta \theta_{6}\;=\;3$° and $\Delta \theta_{8}=0$°.

**Figure 12:**
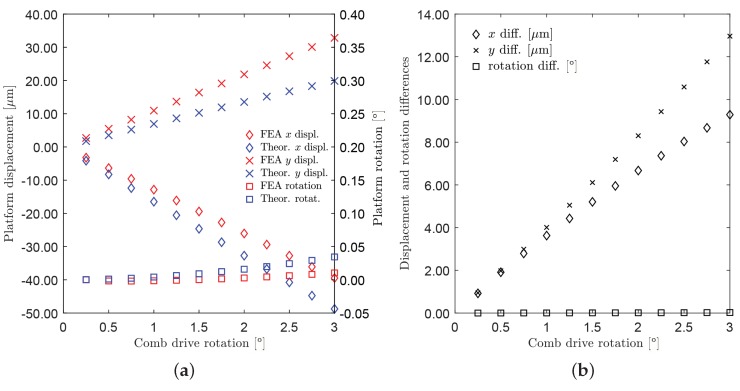
Case of non-uniform rotations ($\Delta \theta_{2}\;=\;-\Delta \theta_{6}\;=\;\Delta \theta \;,\Delta \theta_{8}\;=\;0$). (**a**) Displacements of the platform tip along *x*-axis and *y*-axis (diamond and cross markers, left-hand side axis) and rotations of the platform (square markers, right -and side axis) as a function of Δθ; red and blue colors refer to FEA and theoretical simulations, respectively. (**b**) Absolute differences between FEA and theoretical simulated values (see the legend).

Figure 11 shows the neutral and the deformed configurations for the case $\Delta \theta_{2}\;=\;-\Delta \theta_{6}\;=\;\Delta \theta \;=\;3$°. The platform rotations $\Delta \theta_{4}$ and the tip displacements (point P), with respect to the assigned comb-drive rotations Δθ, are reported in Figure 12a. When the maximum input rotation Δθ=3° was applied, the platform rotation $\Delta \theta_{4}$ did not rotate beyond 0.04°, while the platform center tip moved about 50 μm from the original position. In fact, for this second case, the translational motion was prevailing over the rotational motion. Figure 12b represents the absolute differences among the results obtained by means of FEA and theoretical simulation. As for the previous case, for a given input rotation Δθ=3° of the moving arms, the methods predicted the platform rotations $\Delta \theta_{4}$ with a difference of about a few thousandths of a degree, while the maximum difference in *x* or *y* linear displacements was about 10 μm.

## 5. Method of Fabrication

Both the platform and microgrippers can be simultaneously fabricated on SOI wafers. The wafers consist of a thick lower silicon layer, usually named the *handle* layer, and of a top silicon layer, usually referred to as the *device* layer. The two layers are separated by a thin silicon oxide layer (buried silicon oxide). Both sides can be etched using semiconductor standard DRIE technology, down to the buried silicon oxide, which acts as etching stop-layer.

**Figure 13:**
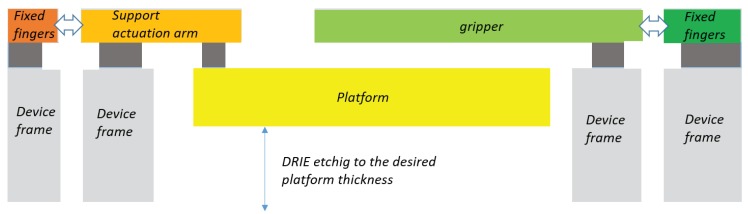
Schematic representation of the structure cross-section.

Thanks to the fabrication process it is possible to obtain, on a single SOI wafer, suspended elements able to move on two parallel layers. With reference to Figure 13, these two layers are separated by the thinner oxide layer (dark grey), which selectively connects the upper parts either to the handle zones (green and orange) or to the mobile platform (yellow). The oxide also provides electrical insulation. The device layer contains some anchored pads (dark green and orange) and most of the mobile suspended elements of the systems: the comb-drives, the electrode pads, the mobile arms of the grippers (light green and light orange).

**Figure 14:**
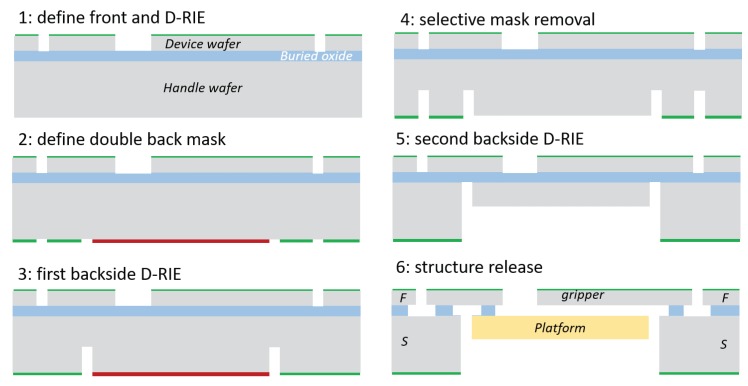
An example of the fabrication sequence and structure section:the actuation finger *F* and the supporting parts *S*.

The handle layer contains the fixed supports on which the upper anchors are attached by means of the oxide middle layer (see the light grey sectional areas in the figure). Furthermore, the lower layer is also used to fabricate the sample platform (yellow cross-sectional area in Figure 13 and Figure 14 (6)).

Etching on the platform area is performed with a limited time interval, while the handle areas remains protected. In fact, the fabrication of the platform (yellow) is planned in such a way that etching is stopped before the oxide stop-layer is reached. Process time depends on platform thickness. Therefore, the platform can move below the device layer, being attached to three RRR arms by means of three clamping elements.

The main steps of fabrication process are illustrated in Figure 14 and are described below.

1. The front side is firstly processed by depositing and patterning a masking layer by means of photolithography. Then, DRIE is performed on the front side (Figure 14(1)), reaching the buried silicon oxide.
2. Once the SiO had been reached, the same mask is deposited and patterned on the wafer backside. Before etching the backside, a second masking layer is deposited and patterned over the first one, to perform a two-step backside DRIE (Figure 14(2)).
3. Next, the first step is etched (Figure 14(3)).
4. Then, the second mask is removed (Figure 14(4)).
5. Once the second mask is removed, the etching is completed once the buried oxide from the backside is reached (Figure 14(5)).
6. The final isotropic etch is performed to partially remove the buried silicon oxide and to release the moving parts.

## 6. Discussion

The development of a new microsystem based on a single wafer becomes a rather ambitious problem when several revolute joints and degrees of freedom are required. The solution presented in this paper owes much to the adoption of flexure hinges with an elastic curved beam (lumped compliance) and of a particular sequence of process steps. Before starting the expensive DRIE fabrication, it was therefore convenient to simulate the systems and optimize the geometrical and physical properties. The previous sections have presented both the fabrication and simulation methods that will be now briefly discussed.

### 6.1. About the Simulation

**Figure 15:**
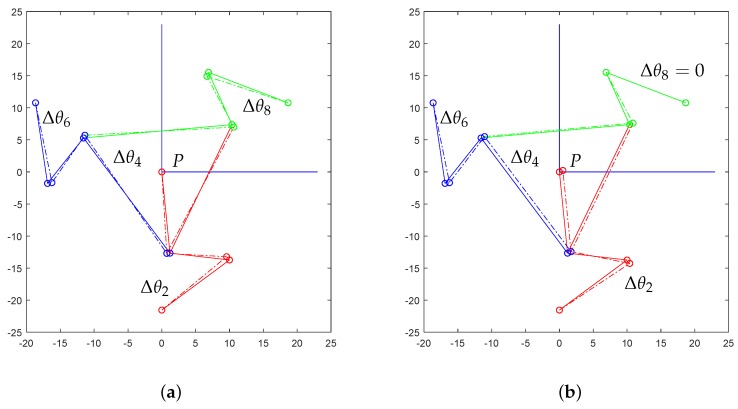
Configuration analysis via theoretical model: in the first case (**a**), the center tip *P* of Platform 4 does not move significantly, while the platform rotates by $\Delta \theta_{4}$; in the second case (**b**), the platform rotation $\Delta \theta_{4}$ is almost null, while the platform center tip *P* moves toward the fixed revolute pair 1–8.

The mechanical structure of the developed microsystem was based on lumped compliance, and so, a group of flexure hinges was introduced, while the other parts were more rigid and maintained their original undeformed shapes with a good approximation. Flexures were used to connect two adjacent rigid parts, say $C_{i}$ and $C_{j}$, and so, their relative motion would be defined by the position of the relative center of rotation $P_{ij}$. Considering the simulation results, a correct definition of the centers $P_{ij}$ for any revolute pair i−j played a fundamental role in applying the above mentioned joint-replacement method of design. In this investigation, these points have been determined according to recent theoretical models [42]. Thanks to these methods, the developed microsystem and the corresponding PRBM have shown a good match in the obtained results. In fact, considering the two analyzed cases, the differences of the results obtained by means of the theoretical model and the FEA were negligible (see Figure 11 and Figure 12). The two cases can be interpreted by using more in detail the results obtained by means of the theoretical simulation as reported in Figure 15. In the first case, the three moving links 2, 6, and 8 were rotated by the same angle. Therefore, considering the structure symmetry, this coordinated actuation had the effect of inducing a rotation on the platform around the platform center *P*, as illustrated in Figure 15a. In the second case, link 8 was assumed to be non-actuated, while the other two moving links rotated in opposite directions by the same angle. Once again, by virtue of the symmetric geometry of the mechanical structure of the device layer, the platform did not significantly rotate, while it moved toward the center of the fixed hinge 8. Of course, point *P* moved accordingly along the same direction, as illustrated in Figure 15b.

### 6.2. About the Process

**Figure 16:**
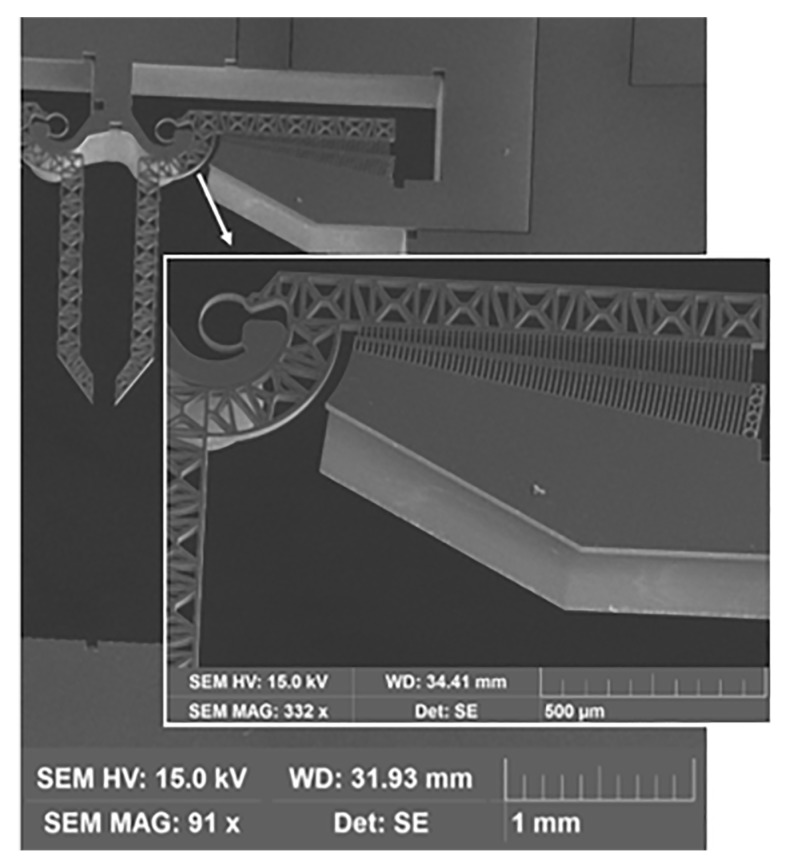
Scanning electron microscope (SEM) image of a fabricated microgripper device block.

All the process steps have been tested by the research group in the recent past; therefore, the process parameters are likely to be quickly tuned. Some encouraging results were reported in [44]. Several example of complex device layers have been obtained, such as the fabricated microgripper device block reported in Figure 16.

## 7. Conclusions

The new microsystem concept presented in this paper is promising for a large variety of micromanipulation and lab-on-chip applications. The system consists of a MEMS-technology-based device with four DoFs, three of which were for the platform mobility and one for the grasping action of the microgrippers. The fabrication process relies on a procedure that has been already tested by the research group, with satisfying results, while the geometry has been validated by two simulation methods, one based on rigid-body mechanisms theory and one based on the FEA of compliant mechanisms. The whole microsystem can be obtained with a single wafer, exploiting the characteristic properties of SOI wafers.

## 8. Patents

This article presents a new concept microsystem that has been registered at the Italian Patent Office on 13 March 2019. The paper illustrates the characteristics of the new system and shows possible ways to exploit the invention. For more information concerning the above-mentioned patent, the reader can refer to the following essential data.

Bagolini, A., Belfiore, N.P., Micromanipulator and method to fabricate this micro-manipulator (in Italian), *Micromanipolatore e metodo per la realizzazione di tale micro-manipolatore*, Ufficio Italiano di Brevetti e Marchi (UBIM), Ministero dello sviluppo economico, Domanda Numero 102019000003941, March 13th, 2019, property of Fondazione Bruno Kessler, Trento.

## Abbreviations

The following abbreviations are used in this manuscript:

CSFH	conjugate surfaces flexure hinge
DoF	degrees of freedom
DRIE	deep-reactive ion etching
FEA	finite element analysis
SOI	silicon on insulator
